# Artificial Intelligence-Enabled Bioengineering of Extracellular Vesicle Platforms in Cardiovascular Medicine

**DOI:** 10.3390/bioengineering13050573

**Published:** 2026-05-19

**Authors:** Nurittin Ardic, Rasit Dinc

**Affiliations:** 1 Med-International UK Health Agency Ltd., Nuneaton CV11 6LT, UK; nurittinardic@yahoo.com; 2 INVAMED Medical Innovation Institute, One World Trade Centre, New York, NY 10007, USA

**Keywords:** extracellular vesicles, artificial intelligence, bioengineering, microfluidics, biosensors, precision medicine

## Abstract

Extracellular vesicles (EVs) hold significant potential in cardiovascular diagnosis and treatment. However, their clinical applications are limited by challenges such as isolation efficiency, subpopulation heterogeneity, analytical standardization, and manufacturing scalability. Artificial intelligence (AI) and machine learning (ML) offer a computational framework to address these constraints through data-driven platform engineering. This review examines AI-assisted strategies in three interconnected EV platform pillars in cardiovascular medicine. These include: (i) isolation and processing platforms where ML algorithms optimize microfluidic separation and improve signal accuracy; (ii) analytical and diagnostic platforms where deep learning supports single vesicle phenotyping, multi-omics biomarker engineering, and biosensor interpretation; and (iii) therapeutic and manufacturing platforms where AI guides cargo loading, biodistribution estimation, and process control. We also assess key translational challenges, including MISEV2023 compliance, dataset bias, reproducibility, and regulatory alignment. This review positions artificial intelligence as the fundamental layer of the EV bioengineering process, providing a structured framework for advancing EV-based cardiovascular platforms from laboratory research to clinical application.

## 1. Introduction

Extracellular vesicles (EVs) are lipid bilayer-enclosed particles carrying proteins, nucleic acids, lipids, and metabolites. They mediate intercellular communication across the cardiovascular system, playing a role in thrombosis, inflammation, angiogenesis, and tissue remodeling [1,2]. Their molecular cargo reflects the pathological state of the host cells, positioning EVs as candidate biomarkers and therapeutic vectors for conditions ranging from acute coronary syndrome to heart failure and peripheral vascular disease [3,4,5]. In addition, both naturally occurring and engineered extracellular vesicles play an increasingly important role in the pathogenesis and treatment of atherosclerosis, a central disease process in cardiovascular medicine [6,7]. Circulating EVs derived from platelets, endothelial cells, and leukocytes carry disease-specific signatures, including miR-126, miR-21, tissue factor, and phosphatidylserine. These are related to cardiovascular risk and treatment response [3].

However, translating this biological potential into clinically engineered platforms has been challenging. Natural EVs are heterogeneous in terms of size (30–1000 nm), cellular origin, and molecular composition. No single isolation method provides the combination of high yield, high purity, and scalability required for clinical use [8,9]. Characterization data are high-dimensional and poorly standardized across laboratories, and manufacturing lacks the reproducibility demanded by regulatory bodies [10,11]. These represent engineering design constraints and require engineering solutions.

**Figure 1:**
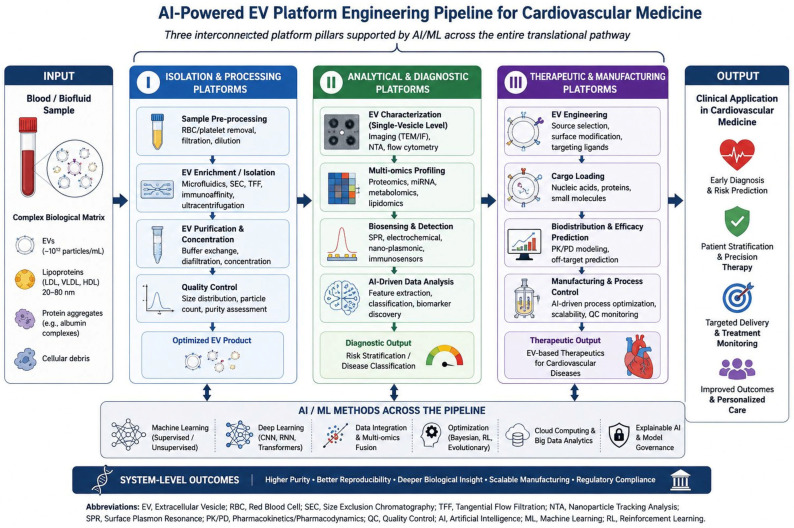
AI-enabled extracellular vesicle (EV) platform engineering framework for cardiovascular medicine. The figure summarizes three interconnected platform pillars involved in cardiovascular EV translation: (I) isolation and processing platforms, (II) analytical and diagnostic platforms, and (III) therapeutic and manufacturing platforms. AI and machine-learning methods support all stages, including optimization, classification, quality control, and translation integration. The framework emphasizes the progression from complex biological inputs to clinically applicable cardiovascular applications.

**Figure 2:**
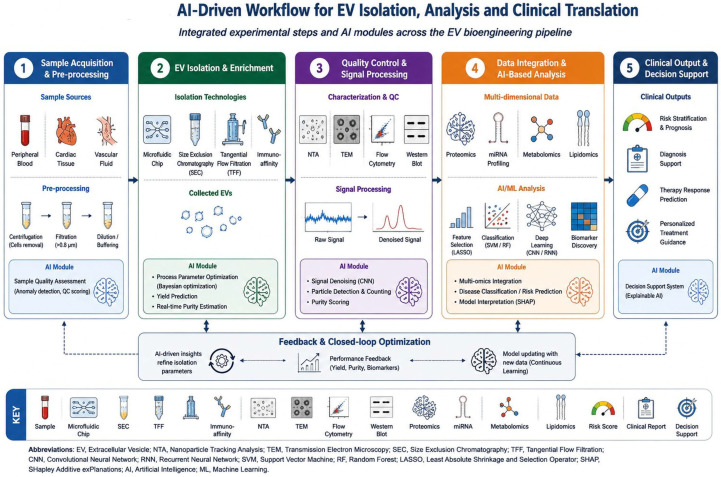
AI-enabled workflow for extracellular vesicle isolation, analysis, and clinical application. The figure illustrates a sequential five-step workflow integrating experimental EV processing with AI-assisted analytical modules. The pipeline includes sample acquisition and preprocessing, EV isolation and enrichment, quality control and signal processing, multi-omics data integration, and AI-based analysis and clinical decision support. Feedback and closed-loop optimization modules ensure continuous refinement of platform performance and biomarker interpretation.

**Figure 3:**
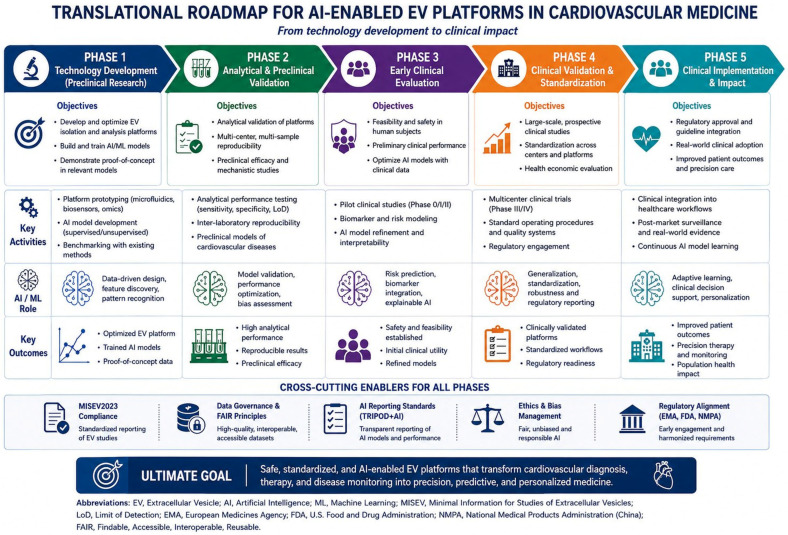
Translation roadmap for AI-enabled extracellular vesicle platforms in cardiovascular medicine. The figure illustrates five sequential translation phases: technology development, analytical validation, early clinical evaluation, clinical standardization, and healthcare implementation. Intersecting facilitators, including MISEV2023 compliance, FAIR data governance, AI reporting standards, ethical frameworks, and regulatory compliance, support all phases of the translation pathway. The roadmap is based on the three platform pillars presented in Figure 1 and outlines the progression toward clinically viable AI-enabled EV systems.

AI provides a unifying computational layer to address these constraints along the EV bioengineering pipeline. Rather than surveying the breadth of AI applications in EV biology, this review focuses on three engineering platform pillars that define the translational pathway from laboratory-scale EV research to clinical cardiovascular application. These include (i) isolation and processing platforms, (ii) analytical and diagnostic platforms, and (iii) therapeutic and manufacturing platforms. To conceptualize the overall architecture of the platform, Figure 1 summarizes three interconnected engineering pillars supporting AI-enabled cardiovascular EV translation. For each pillar, we evaluate specific AI/machine learning techniques that enhance system-level optimization, identify existing engineering bottlenecks, and assess readiness for clinical translation. The integrated experimental and computational workflow is further summarized in Figure 2. We conclude with a translational roadmap (Figure 3) integrating MISEV2023 standardization, regulatory convergence, and phased clinical validation. We first examine the engineering constraints that define EV isolation and processing platforms, as these upstream steps determine the quality of all downstream analytical and therapeutic applications.

### Translational Workflow Overview

A practical translation workflow for AI-powered EV platforms involves five sequential stages: (1) sample acquisition and preprocessing, (2) EV isolation and enrichment, (3) quality control and signal processing, (4) multidimensional data analysis and AI-based interpretation, and (5) clinical output generation, including risk estimation or therapeutic design. Each stage imposes specific engineering constraints and computational requirements, and performance in subsequent stages is critically dependent on data quality in previous stages. This step-by-step framework forms the basis of the platform-oriented analysis presented in the following sections.

## 2. Pillar I: EV Isolation and Processing Platforms

### 2.1. Engineering Design Constraints in Cardiovascular EV Isolation

Isolating cardiovascular EVs from blood samples presents a significant platform engineering challenge. Target EVs are found within a highly complex biological matrix containing approximately 10^12^ particles per milliliter, along with lipoproteins of similar size (LDL: 20–25 nm; VLDL: 30–80 nm), protein aggregates (e.g., albumin complexes), and cellular debris. Ultracentrifugation, the traditional workhorse method, subjects samples to extreme gravitational forces (≥100,000× *g*). This process co-pellets contaminants, resulting in preparations with particle-to-protein ratios far below the MISEV2023 recommendations for high-specificity EV isolation [9,12].

Alternative approaches present trade-offs. Size-exclusion chromatography increases purity but reduces throughput. Tangential flow filtration enables scalable processing but introduces membrane-dependent biases. Immunoaffinity capture provides high specificity for known subpopulations but fails to capture novel or poorly characterized EV subtypes [8,13].

In cardiovascular applications, the clinically relevant EV fraction may represent less than 1% of total circulating vesicles. Therefore, these limitations are not only technical but also platform-defining limitations. Table 1 provides a comparative summary of current EV insulation techniques and their engineering advantages and disadvantages.

This comparison underscores the need for AI-powered platform optimization, highlighting that no single method simultaneously meets the requirements for purity, scalability, and discovery.

From a translational engineering perspective, currently no single isolation method meets all clinical requirements. Ultracentrifugation offers scalability but suffers from low specificity due to the co-isolation of lipoproteins and protein aggregates. Size exclusion chromatography improves purity (particle-to-protein ratio typically > 10^10^) but is limited in efficiency [8,9,10]. Tangential flow filtration supports scalable processing but introduces variability (40–80%) in membrane-bound recovery. Immunoaffinity capture provides high specificity (>90% target enrichment) but is limited to predefined EV subpopulations and lacks exploratory capability. For cardiovascular applications where clinically relevant EVs may represent less than 1% of total vesicles, these trade-offs remain a central bottleneck.

### 2.2. Microfluidic Lab-on-Chip Architectures

Microfluidic lab-on-chip (LoC) devices address many of these limitations by providing precise, programmable control over fluid dynamics, surface interactions, and separation forces at the microscale [14,15].

Contemporary LoC architectures integrate multiple physical principles in cascaded configurations. Deterministic lateral displacement (DLD) arrays initially perform size-based pre-enrichment. Acoustic wave stages then improve separation based on density and compressibility. Finally, immunoaffinity capture sites selectively isolate target EV subpopulations based on surface marker expression [16,17].

This cascaded design achieves over 90% particle purity and over 70% recovery rates from whole blood within 30 min. These performance metrics significantly surpass those of conventional isolation methods [13].

For cardiovascular point-of-care applications, minimum sample volume requirements (as low as 50–100 μL) enable EV profiling from routine venous puncture without the need for specialized processing infrastructure. However, these systems are governed by a high dimensional and interdependent parameter space. A single three-stage device may contain 15–20 independent variables, including flow rates, channel geometries, pillar spacing, acoustic frequencies, electric field strengths, antibody surface densities, and incubation times. These variables exhibit complex nonlinear interactions, making manual optimization impractical in clinical development timescales [13,18].

### 2.3. ML-Driven Platform Optimization

Machine learning (ML) algorithms are well-suited for navigating the high-dimensional design space of microfluidic EV platforms.

Bayesian optimization is widely used for this purpose. It generates probabilistic surrogate models of the objective function and selects experiments that maximize the expected recovery. In microfluidic EV capture design, this approach converges to device configurations that outperform manually designed systems while reducing the number of experimental iterations from hundreds to below 30 [18].

Gaussian process regression further models the relationship between device parameters and EV recovery efficiency with quantified uncertainty. This enables the determination of robust operating ranges that tolerate the manufacturing variability required for clinical-grade systems [19].

Reinforcement learning represents an emerging frontier. These agents enable adaptive and potentially patient-specific isolation protocols by iteratively adjusting flow rates and field strengths in real-time during device operation [20]. In cardiovascular applications, machine learning-optimized platforms have demonstrated selective capture of platelet-derived microvesicles (CD41^+^/CD62P^+^ EVs) while excluding lipoproteins with similar hydrodynamic diameters. This level of selectivity is still difficult to achieve with conventional methods [15,20].

Transfer learning further accelerates the platform development process. Models trained on well-characterized EV subtypes (e.g., tumor-derived exosomes) can be adapted to cardiovascular targets with limited data, thus mitigating the cold start problem in the clinical biomarker development process.

However, it is important to distinguish between experimentally validated applications and conceptual applications. While Bayesian optimization and supervised learning approaches demonstrate measurable improvements in EV recovery and selectivity, reinforcement learning-based adaptive systems and fully autonomous optimization processes largely remain in the proof-of-concept stage.

### 2.4. AI-Enhanced Signal Processing and Quality Control

After isolation, detection sensitivity is generally limited by low signal-to-noise ratios. This poses a problem, especially when target cardiovascular EVs are present in low concentrations (10^4^–10^6^ particles/mL) against a background containing abundant non-target particles.

Convolutional neural networks (CNNs) trained on labeled nanoparticle tracking analysis (NTA) images can distinguish true EV scattering events from Brownian motion artifacts. These increase counting accuracy by 15–40% compared to conventional threshold-based methods [19,21].

Wavelet-based spectral filtering further improves signal quality. When applied to surface plasmon resonance (SPR) biosensor data, it eliminates electronic noise while preserving the fast-coupling kinetics required for real-time EV quantification [17]. These computational approaches are particularly important for miniaturized bedside care platforms. Hardware limitations such as reduced optical path length, lower-cost detectors, and shorter integration times impose fundamental constraints on signal quality. In such systems, performance improvements depend primarily on software-level intelligence. Figure 2 illustrates the integrated experimental and computational workflow linking EV isolation, signal processing, multi-omic integration, and AI-assisted clinical interpretation.

Following the optimization of isolation and signal quality, the next translational challenge is to convert high-dimensional EV measurements into biologically and clinically meaningful analytical outputs.

In summary, EV isolation remains a fundamental engineering bottleneck, and AI-powered optimization offers measurable improvements, but full clinical standardization has not yet been achieved.

## 3. Pillar II: Analytical and Diagnostic Platforms

### 3.1. AI-Driven EV Subpopulation Classification

Resolving cardiovascular EV subpopulations from high-dimensional datasets requires computational methods that go beyond traditional manual gating. A single plasma sample analyzed by high-parameter nano-flow cytometry can generate 10^5^–10^6^ individual EV measurements. Each vesicle can be identified by 10–20 features, including size, refractive index, and multiple fluorescence channels [22,23].

Unsupervised clustering methods such as k-means, density-based spatial clustering (DBSCAN), and self-organizing maps can identify EV subsets without making prior assumptions about population structure. These approaches often reveal cardiovascularly significant clusters that manual gating cannot detect [19].

Supervised classifiers, including random forests, gradient-enhanced decision trees, and support vector machines, can then assign individual vesicles to functional categories such as prothrombotic, proangiogenic, and proinflammatory states. These classifications are based on integrated surface marker and cargo profiles and have achieved cross-validation accuracy of 85–95% in recent studies [3,24].

Ensemble methods combining multiple classifiers generally outperform individual models. This advantage is particularly important in heterogeneous clinical cohorts where within-patient variability is high.

### 3.2. Single Vesicle Analysis and Deep Learning

Single vesicle characterization technologies, including nanoflow cytometry, single-particle interferometric reflection imaging (SP-IRIS), surface plasmon resonance microscopy (SPRm), and atomic force microscopy (AFM), generate highly detailed datasets requiring equally advanced computational analysis [22,25,26].

Deep learning architectures are particularly well suited for this task. Autoencoders and variational autoencoders (VAEs) can learn compressed latent representations of high-dimensional single vesicle datasets. These models map a continuous phenotypic landscape rather than forcing vesicles into rigid categories [23].

This approach enables the identification of previously unrecognized vesicle subtypes. For example, rare tissue factor-positive endothelial EVs recently associated with thrombotic risk may constitute less than 0.1% of the total EV population and may not be detectable using aggregate characterization methods. Graph neural networks (GNNs) and attention-based transformer models are emerging as additional tools for learning phenotype-function relationships from integrated phenotypic and functional data. These models can predict the biological effects of novel EV subsets without requiring direct functional testing, thus accelerating the screening of cardiovascular EV biomarker candidates [21,27,28].

### 3.3. Engineering Multi-Omics Biomarker Panels

Cardiovascular EV content encompasses multiple molecular layers, including proteomics, transcriptomics, lipidomics, and metabolomics. The proteomic content includes over 4000 identified proteins such as troponins, natriuretic peptides, and matrix metalloproteinases. The transcriptomic content includes miR-126, miR-21, miR-155, and long non-coding RNAs, while the lipidomic content includes ceramides, phosphatidylserine, and sphingomyelins [29,30].

Each omics modality provides only a partial molecular perspective; integration is essential to create clinically applicable biomarker panels. Recent studies have shown that AI-integrated multi-omics EV analysis significantly improves biomarker discovery and reproducibility in cardiovascular disease [31]. However, most current applications remain retrospective or offline analytical tools, while the real-time clinical application of AI-powered EV diagnosis is still limited.

AI-powered feature selection methods such as LASSO regularization, elastic net, mutually informative approaches, and recursive feature elimination can identify the most informative molecular features while reducing overfitting in the high-dimensional, small-sample environment typical of clinical EV studies [27].

The engineering goal is to reduce thousands of candidate features to compact biomarker panels containing fewer than 10 markers that can be implemented in scalable diagnostic platforms. This is a constraint-based design problem and is well suited to machine learning optimization [32].

Deep learning fusion architectures can integrate proteomic and transcriptomic EV data with clinical variables such as demographic data, imaging, and electrocardiography. These models capture nonlinear interactions that traditional linear approaches might miss, and recent reports have shown area under the curve (AUC) values of 0.90–0.96 in predicting major adverse cardiovascular events (MACE) [21,33]. Interpretability is essential. Methods such as SHAP (Shapley Additive Explanations) and LIME (Local Interpretable Model-agnostic Explanations) increase clinician confidence and support regulatory acceptance by decomposing model outputs into trait-level contributions [19].

### 3.4. AI-Powered Biosensor and Point-of-Care Platforms

Transforming EV biomarker panels into viable cardiovascular diagnostics requires biosensor platforms with sufficient sensitivity to detect target EVs at clinically relevant concentrations (10^4^–10^8^ particles/mL).

Various nano-biosensor methods, including surface plasmon resonance (SPR), localized SPR (LSPR), electrochemical impedance spectroscopy (EIS), field-effect transistors (FETs), and quartz crystal microbalance (QCM) systems, now provide the necessary analytical performance [23,25].

Artificial intelligence contributes to biosensor platform engineering at multiple levels. Generative models can computationally design recognition elements such as aptamers, nanobodies, and peptides optimized for target EV surface markers. Finite element modeling, when combined with machine learning, can predict optimal sensor surface architectures. Pattern recognition algorithms can then interpret multiple biosensor signals in real time [34].

For continuous cardiovascular monitoring, microfluidic biosensors combined with long short-term memory (LSTM) recurrent neural networks can track temporal EV dynamics, including changes in concentration, size distribution, and surface marker expression over hours to days. These systems can detect temporal signatures of acute cardiovascular events that single time-point measurements cannot capture [14,19].

Smartphone-connected point-of-care (POC) systems represent the translation frontier. By integrating miniaturized microfluidic isolation, nano-biosensor detection, and on-device ML inference, these platforms support decentralized cardiovascular screening. Edge computing further enhances privacy protection in clinical settings by enabling real-time processing without cloud reliance [32] (Figure 2). Beyond diagnostic applications, these same computational principles extend directly to therapeutic engineering and the scalable production of EV-based cardiovascular platforms.

In summary, AI improves EV analytical resolution and biomarker discovery, but its real-time clinical integration remains limited.

## 4. Pillar III: Therapeutic and Manufacturing Platforms

### 4.1. AI-Powered Cargo Loading and Surface Engineering

Engineering EVs as therapeutic delivery vehicles for cardiovascular repair requires precise control over two interdependent variables: internal cargo composition and external surface functionalization.

Current loading strategies include endogenous, exogenous, and hybrid approaches. Endogenous loading relies on genetic modification of parent cells to overexpress therapeutic RNAs or proteins. Exogenous loading utilizes methods such as electroporation, sonication, extrusion, or chemical transfection of purified EVs. Hybrid strategies combine elements of both approaches. Each strategy presents optimization challenges that expand combinatorially with cargo type, EV source, and target tissue [35,36].

Artificial intelligence shifts this process from empirical screening to guided design. Predictive models trained on cargo physicochemical properties such as molecular weight, charge, and hydrophobicity, EV membrane composition, and processing parameters can predict loading efficiency. This can eliminate 70–80% of unpromising experimental conditions and concentrate wet lab studies on the most suitable candidates [21].

Generative adversary networks (GANs) and variational autoencoders can further explore the design space for synergistic multiple cargo combinations. For example, these models can identify miRNA–protein pairs with additive cardioprotective effects that are impractical to discover alone through conventional experimental screening [19].

Surface engineering presents a parallel challenge. Molecular dynamics (MD) simulations combined with deep learning can predict EV–cell membrane interactions at near-atomic resolution. These models support the rational selection of targeting ligands, including heart-oriented peptides and anti-VCAM-1 aptamers, and help optimize ligand density and orientation for better uptake by target cardiovascular cell types [21,37].

### 4.2. AI-Assisted Cardiovascular Repair Applications

Mesenchymal stem cell-derived EVs (MSC-EVs), endothelial progenitor cell-derived EVs, and cardiac progenitor cell-derived EVs have demonstrated preclinical therapeutic efficacy in myocardial infarction, ischemia-reperfusion injury, atherosclerosis, and vascular remodeling. Their effects occur via anti-inflammatory, pro-angiogenic, and anti-apoptotic pathways [38,39,40,41].

AI-assisted cargo analysis helps to identify the molecular mediators responsible for these therapeutic effects. These include specific miRNAs such as miR-21, miR-132, and miR-210, as well as proteins like VEGF, HGF, and SDF-1, and selected lipid types. This approach supports the design of defined EV-mimetic formulations with optimized therapeutic indices, rather than relying on unfractionated secretome preparations [19,42].

Unlike previous EV therapeutic frameworks, the emphasis here is on AI-guided design and predictive optimization rather than purely empirical engineering.

The integration of EV therapeutics with cardiovascular device engineering represents a platform-level innovation. EV-functionalized vascular stents can deliver a local anti-inflammatory load at implantation sites. EV-coated grafts can promote endothelialization while suppressing neointimal hyperplasia. EV-loaded heart valve prostheses can modulate local immune responses and reduce calcification. In these environments, AI-optimized EV formulations can be adapted to address device-specific failure modes [2,43]. This concept aligns with emerging EV-based device engineering strategies for vascular healing and drug delivery.

### 4.3. Manufacturing Process Control and Scale-Up

The transition from laboratory-scale EV production to clinical production requires platform-level solutions for consistency, scalability, and regulatory alignment.

Current EV production workflows exhibit significant batch-by-batch variations in particle concentration, size distribution, protein content, and functional potential. Reported coefficients of variation for particle concentration can reach 20–50%, which is unacceptable for pharmaceutical-grade products [11,13].

AI-powered process control can address this problem through three complementary mechanisms.

First, digital twin models of bioreactor systems can be continuously updated with real-time sensor data, including dissolved oxygen, pH, glucose consumption, and EV release rates. These models can predict batch results before harvest and suggest corrective parameter adjustments to maintain production characteristics [19].

Secondly, automated image analysis of transmission electron microscopy (TEM) and cryoelectron microscopy (cryo-EM) quality control samples using CNNs can provide rapid and objective morphological assessment. This reduces reliance on subjective manual scoring. Thirdly, multivariate statistical process control (MSPC) integrated with machine learning can detect subtle process deviations associated with downstream potential loss. This enables preventive intervention instead of retrospective batch rejection. The integration of automated microfluidic production lines with AI-based quality assurance systems represents one of the most direct pathways towards GMP-compliant and scalable EV production for cardiovascular applications. However, technical feasibility alone is not sufficient for transitioning to clinical practice; these platforms must also meet standards for reproducibility, data transparency, and regulatory acceptance.

To summarize the AI/machine learning methodologies and their corresponding engineering applications discussed across the three platform columns, Table 2 provides an integrated overview relating computational techniques to cardiovascular use cases.

## 5. Translational Challenges and Standardization

### 5.1. MISEV2023 Compliance for AI-Processed EV Data

MISEV2023 defines consensus requirements for EV characterization. These include morphology confirmation by electron microscopy, nanoparticle tracking analysis (NTA) or size distribution analysis by dynamic light scattering, and protein marker profiling with at least three positive and two negative markers [10,12].

When AI algorithms are applied to EV datasets, additional transparency requirements arise that are not fully addressed by current guidelines. To ensure computational reproducibility, studies should report model architecture, training data features, preprocessing steps, hyperparameter settings, software versions, code availability, and performance metrics such as accuracy, sensitivity, specificity, and AUC, along with confidence intervals [29,44]. In addition, standardized reporting of AI models (including architecture, training datasets, preprocessing steps, and validation metrics) should be considered essential for reproducibility, consistent with emerging frameworks such as TRIPOD + AI for clinical prediction models. Therefore, we recommend adding a dedicated section to AI/machine-learning reporting standards for EV research to future MISEV updates. Such guidance could parallel frameworks like TRIPOD + AI for clinical predictive models and help ensure that AI-powered EV studies meet the same transparency standards expected for experimental workflows.

### 5.2. Dataset Bias, Generalizability, and Federated Learning

AI models trained on single-center, demographically narrow, or platform-specific datasets may generalize poorly to broader cardiovascular populations. This is particularly important because disease prevalence, presentation, and outcomes vary by age, gender, ethnicity, and geography [19,27].

Therefore, standardized pre-analytic protocols are essential for generating high-quality multi-center datasets. These protocols should define variables such as blood collection tube type, processing time, centrifugation conditions, and storage temperature and duration [8].

Federated learning offers a privacy-preserving strategy for generating diverse training datasets without centralizing sensitive patient information. By training models on distributed cardiovascular cohorts, it can enhance generalizability while maintaining data protection.

Augmenting synthetic data using GANs can further support the representation of undersampled demographic groups. However, such synthetic data still need to be validated against real-world clinical datasets. For any AI-assisted cardiovascular EV diagnosis, external validation on demographically representative test sets should be considered a minimum requirement. Performance should also be reported transparently across clinically relevant subgroups.

### 5.3. Regulatory Convergence and Clinical Validation Pathways

AI-powered EV platforms are situated at the intersection of three regulatory areas: in vitro diagnostics (IVD), biological therapeutics, and Software as a Medical Device (SaMD).

Several regulatory frameworks are important. These include the FDA’s evolving approach to AI/ML-based SaMD, including a proposed predetermined change control scheme for adaptive algorithms, the European Union’s In Vitro Diagnostics Regulation (IVDR), and the MHRA’s guidance on AI in medical devices. While these frameworks differ, they share overlapping expectations regarding clinical validation, algorithm transparency, and post-market surveillance [11,23,35].

Additional requirements apply to EV-based therapeutics. These include manufacturing consistency, efficacy testing, and biodistribution studies.

Figure 3 presents a phase translational roadmap for the clinical development and implementation of AI-enabled EV platforms in cardiovascular medicine.

Regulatory science specifically tailored to AI-engineered EV products remains an urgent priority, especially in the context of lifecycle-based validation and adaptive algorithm governance frameworks [45]. This includes frameworks capable of addressing adaptive algorithms, biological variability, and convergent device-biological-software classification. With these translational requirements defined, the field is now ready to move towards more adaptable, personalized, and clinically integrated EV platform systems.

## 6. Future Directions

Three platform-level developments are likely to shape the next phase of AI-powered EV bioengineering in the field of cardiovascular diseases.

First, patient-specific digital twins could become a significant translational tool. By integrating circulating EV molecular profiles with cardiovascular imaging, hemodynamic modeling, and electronic health record data, these systems can support real-time prediction of disease progression and treatment response. They can also simulate how engineered EV therapeutics will interact with an individual patient’s vascular anatomy and immune environment prior to clinical application [19].

Second, personalized EV therapeutics represent a longer-term but highly attractive goal. These will involve custom-designed vesicles engineered from patient-specific multi-omics data using AI-driven cargo selection and surface optimization. Advances in iPSC-derived EV manufacturing, automated closed-loop system fabrication, and AI-powered real-time quality control are making this vision increasingly likely [21,43].

Thirdly, EV-sensing biosensors can be integrated with implanted cardiovascular devices such as stents, pacemakers, and prosthetic valves. This could enable closed-loop monitoring systems that sense local EV dynamics at the device-tissue interface. Such platforms could provide early warning of thrombosis, infection, or structural degeneration and even trigger automated therapeutic EV release in future designs [2,23].

All these future directions are dependent on the same engineering fundamentals discussed throughout this review, namely isolation, analysis, and manufacturing. Continuous attention will also be needed to standardization, validation, and regulatory alignment issues.

Taken together, these developments reinforce the need for a unified platform-based framework that integrates engineering, computational, and clinical translation.

## 7. Conclusions

AI is reshaping the bioengineering of extracellular vesicle platforms along three fundamental pillars defining cardiovascular translation.

In isolation and processing, machine learning-optimized microfluidic architectures can deliver higher separation performance than can be achieved with manual design alone. In analytical diagnostics, deep learning transforms raw EV data into clinically meaningful cardiovascular risk information by providing single vesicle resolution, multi-omics integration, and real-time biosensor interpretation. In therapeutic and manufacturing platforms, AI-powered process control and digital twin models help address batch variability and scalability constraints that have historically limited regulatory progress for EV-based products.

Realizing this potential will require sustained attention to three intersecting priorities. These include MISEV2023-compliant reporting of AI methodologies with full computational transparency, reducing dataset bias through unified multi-center strategies, and developing convergent regulatory frameworks for AI-engineered EV products spanning diagnostic, therapeutic, and software domains. The roadmap proposed in this review is structured with a focus not only on biological events but also on tangible, buildable platform systems. Therefore, it offers a practical framework for researchers, engineers, and clinicians working to advance EV-based cardiovascular medicine from concept to clinical application.

## Figures and Tables

**Table 1:** Comparison of EV isolation techniques in cardiovascular applications.

Method	Yield	Purity	Scalability	Clinical Readiness	Key Limitations
Ultracentrifugation	High	Low	High	Moderate	Lipoprotein contamination
Size exclusion chromatography (SEC)	Moderate	High	Low	Moderate	Low throughput
Tangential flow filtration (TFF)	High	Moderate	High	High	Membrane variability
Immunoaffinity capture	Low-Medium	Very high	Low	Emerging	Bias towards known markers

**Table 2:** AI/machine-learning approaches across three EV platform pillars.

Platform Pillar	Pipeline Stage	AI/ML Technique	Engineering Application	CV-Relevance	Key References
I. Isolation	Microfluidic optimization	Bayesian optimization, RL, surrogate models	Parameter tuning, design space exploration	Platelet-EV capture from whole blood	[18,20]
I. Processing	Signal processing	CNNs, wavelet filtering	Noise reduction, artifact removal	Low volume cardiovascular EV detection	[17,21]
II. Analytics	Subpopulation classification	Clustering, RF, autoencoders, GNNs	Single EV phenotyping, phenotype-function matching	ACS risk stratification, HF severity	[22,23,26]
II. Analytics	Biomarker engineering	LASSO, elastic net, deep fusion, SHAP/LIME	Multi-omics feature selection, explainability	MACE prediction panels (AUC 0.90–0.96)	[27,32,33]
II. Diagnostics	Biosensor design	Generative models, LSTM RNNs, edge ML	Aptamer design, real-time monitoring, POC inference	POC cardiac diagnostics, temporal EV monitoring	[14,25,34]
III. Therapeutic	Cargo and surface optimization	GANs, VAEs, MD + deep learning	Loading efficiency, cardiac-homing ligand design	Targeted EV delivery, EV–device integration	[21,37]
III. Manufacturing	Process control and QC	Digital twins, CNN image analysis, MSPC + ML	Batch consistency, yield optimization, morphology QC	GMP-grade cardiovascular EV production	[13,19]

## Data Availability

The datasets supporting the findings of this review are cited within the article and are available in the referenced original publications.

## Abbreviations

AFM, Atomic Force Microscopy; AI, Artificial Intelligence; ACS, Acute Coronary Syndrome; AUC, Area Under the Curve; CNN, Convolutional Neural Network; CV, Cardiovascular; DBSCAN, Density-Based Spatial Clustering of Noisy Applications; DL, Deep Learning; DLD, Deterministic Lateral Displacement; EIS, Electrochemical Impedance Spectroscopy; EV, Extracellular Vesicle; FET, Field-Effect Transistor; GAN, Generative Adversarial Network; GMP, Good Manufacturing Practices; GNN, Graph Neural Network; HF, Heart Failure; iPSC, Induced Pluripotent Stem Cell; ISEV, International Society for Extracellular Vesicles; IVD, In Vitro Diagnostic; IVDR, In Vitro Diagnostic Regulation; LASSO, Least Absolute Shrinkage and Selection Operator; LIME, Local Interpretable Model-agnostic Explanations; LoC, Lab-on-a-Chip; LSPR, Localized Surface Plasmon Resonance; LSTM, Long Short-Term Memory; MACE, Major Adverse Cardiovascular Events; MD, Molecular Dynamics; MHRA, Medicines and Healthcare Products Regulatory Agency; MISEV, Minimal Information for Studies of Extracellular Vesicles; ML, Machine Learning; MSC, Mesenchymal Stem Cells; MSPC, Multivariate Statistical Process Control; NTA, Nanoparticle Tracking Analysis; POC, Point of Care; QCM, Quartz Crystal Microbalance; RF, Random Forest; RL, Reinforcement Learning; RNN, Recurrent Neural Network; SaMD, Software as a Medical Device; SHAP, Shapley Additive Explanations; SP-IRIS, Single-Particle Interferometric Reflection Imaging; SPR, Surface Plasmon Resonance; SPRm, Surface Plasmon Resonance Microscopy; TEM, Transmission Electron Microscopy; VAE, Variational Autoencoder; VEGF, Vascular Endothelial Growth Factor.
